# Improving Plant Health Through Nutrient Remineralization in Aquaponic Systems

**DOI:** 10.3389/fpls.2021.683690

**Published:** 2021-06-14

**Authors:** Victor P. Lobanov, Doriane Combot, Pablo Pelissier, Laurent Labbé, Alyssa Joyce

**Affiliations:** ^1^Department of Marine Sciences, University of Gothenburg, Gothenburg, Sweden; ^2^PEIMA-INRAe, UE0937, Fish Farming Systems Experimental Facility, Sizun, France

**Keywords:** controlled environment agriculture, aquaponics, nutrient remineralization, solid waste treatment, recirculating aquaculture system, liquid fertilizer

## Abstract

The exploitation of readily bioavailable fish excreta as a source of plant nutrients lies at the cornerstone of aquaponics farming. Research on nutrient cycling in aquaponic systems has devoted considerable attention to the plant uptake of dissolved nutrients in fish excreta, however, the integration of particulate-bound nutrients into downstream hydroponic farming has remained elusive. The high amount of organic carbon present in fish sludge may lead to biofouling if directly incorporated into hydroponic circulation systems, reducing the utility of incorporating fish solids on a large scale. In this study, we implemented a novel treatment system capable of reducing the carbon and nitrogen load of fish solids to produce a liquid fertilizer for a downstream hydroponics unit. Lettuce (*Lactuca sativa*) fertilized with exclusively a commercial nutrient solution, the biofilter effluent (coupled aquaponic system), effluent from the solids treatment system, or the latter two combined were grown in nutrient flow technique gutters downstream of a recirculating aquaculture system stocked with rainbow trout (*Oncorhynchus mykiss*). While crop yields were lower for the aquaponic treatments compared to lettuce grown in a commercial nutrient solution, plant sap analysis demonstrated a contrasting picture with respect to internal nutrient concentrations. Lettuce grown in the commercial hydroponic solution were deficient in several mineral nutrients (Mg, Ca, Na, and Si) nor did they have higher iron concentrations despite the significantly higher EDTA-chelated aqueous iron (460 × greater than other treatments) in the nutrient solution. Nutrient uptake in the rhizosphere was not investigated on a molecular level, although stunted rhizosphere growth in the commercial nutrient solution control suggests a weakened capacity for nutrient uptake in comparison to other treatments. Alongside the remineralization of micronutrients, the solids treatment system addressed the common issue of excess carbon leading to biofouling via a total suspended solids reduction of 87.27% ± 9.95 during the coupled aquaponics cultivation period. Ultimately, these data lead to two important conclusions. Firstly, optimizing nutrient bioavailability is not synonymous to increasing the presence of a nutrient in the water column. Secondly, estimating ideal nutrient solution concentrations involves both preventing nutrient blocking and improving bioavailability.

## Highlights

- The implementation of a low-cost solids treatment system for a freshwater recirculating aquaculture system resulted in a liquid fertilizer more bioavailable to plants than a commercial nutrient solution.
- This study establishes a baseline for macro- and micronutrient acquisition from both soluble RAS waste and fish solids in aquaponics.
- Mineral nutrient uptake in the roots was not found to be linearly correlated to the nutrient solution (background) concentration.
- Aquaponic systems are well adapted to provide most, but not all essential plant nutrients at sufficient concentrations.

## Introduction

In terms of land-use, agricultural production currently occupies half of the world’s habitable land (Ellis et al., 2010; Ritchie and Roser, 2013). A staggering 70% of the global freshwater consumption is currently devoted to agriculture, reaching up to 90% of local supply in some regions (Meier et al., 2018). The need for high nutrient-use efficiency in existing agricultural systems has also risen in importance due to extreme instances of eutrophication from intensive food production as well as potential phosphorus scarcities (Cordell et al., 2009; Metson et al., 2012; Steffen et al., 2015; Schaum, 2018). These challenges have led to the increase of controlled environment agriculture (CEA), a term that covers protected agriculture (e.g., greenhouse, polytunnels, row covers) and technology-integrated crop management systems (e.g., vertical farming, aquaponics) (Benke and Tomkins, 2017; Shamshiri et al., 2018; Hickman, 2019; Yanes et al., 2020). As of 2019, protected agriculture covers 8.83% of all arable land; a figure up from 3.5% in 2016 (Hickman, 2019; Sambo et al., 2019). While CEA platforms are more efficient cultivation strategies, they must contend with significantly higher infrastructure costs in comparison to traditional soil-based agriculture (Lichtenberg et al., 2013; Savvas and Gruda, 2018).

Aquaponics is a potentially interesting growing method that can help mitigate some of the additional infrastructure costs of CEA by coupling hydroponic crop production to recirculating aquaculture systems (RAS) (López-Mosquera et al., 2011; Carvalho et al., 2018; Goddek et al., 2019). In most aquaponics systems, the main aquaculture contribution to hydroponics cultivation is via the biofilter. Biofilters are essential to RAS stability as they remove ammonia that is highly toxic to fish, but they are also the first major N-removal step in coupled aquaponics, with the second being the uptake of nitrogenous species by the crops themselves. The biofilter is simultaneously responsible for the bioconversion of ammonium to nitrate, as well as the reduction of nitrogenous species to nitrogen gas, and removes a significant portion of nitrogen as nitrogen gas (Holl et al., 2011; Yogev et al., 2017). The remaining N-fraction leaves the biofilter largely as nitrate with a minority concentration present as nitrite (Wongkiew et al., 2017). The soluble effluent from a RAS is insufficient to address all plant needs. However, there is significant controversy around the extent to which the nutritional profile should be supplemented with nutrient solution for maximum crop productivity (Endut et al., 2010; Bittsanszky et al., 2016; Ayipio et al., 2019).

While hydroponic nutrient supplementation is an easy way to address specific deficiencies, there is an underexplored potential for the remineralization of RAS solid waste as a parallel waste-to-nutrition pipeline to manage agricultural yields. The first solid waste treatment systems in aquaponics were based on either aerobic or anaerobic microbial digesters to increase the solubility of matrix-bound nutrients, with attention mainly devoted to phosphorus and a few plant-relevant micronutrients (Goddek et al., 2016a, 2018; Khiari et al., 2019). Hitherto unexplored in aquaponics production system are the wide range of aerobic and anaerobic nutrient remineralization systems currently used in municipal wastewater treatment plants worldwide, such as enhanced biological phosphorus removal (EBPR) (Yuan et al., 2012; Cieślik and Konieczka, 2017; Bunce et al., 2018). EBPR has been shown to cheaply and efficiently remineralize the diverse substrate compositions typical of municipal waste (Barnard, 1976; Greaves et al., 1999; Yuan et al., 2012; Cieślik and Konieczka, 2017; Schaum, 2018; Rahman et al., 2019; Takiguchi et al., 2019). Typical to EBPR systems is the enrichment of phosphate accumulating organisms (PAO), which play a pivotal role in simultaneous denitrification, carbon catabolism, as well as cyclic phosphorus uptake and release (Yuan et al., 2012; Stokholm-Bjerregaard et al., 2017). An alternating aerobic-anaerobic environment, typically carried out in a sequential batch reactor (SBR), is essential to the activity of these systems.

While canonical PAOs consist mainly of *Candidatus* Accumulibacter spp., the past decade has shown PAO lifestyles among members of the Actinobacterial genus *Tetrasphaera*; bacteria capable of metabolizing a diverse range of carbon sources (Kristiansen et al., 2013; Marques et al., 2017). Recent studies have furthermore hinted at a relationship between iron and phosphorus in the PAO lifestyle, although the mechanism of action remains unknown (Braak et al., 2016; Bollyn et al., 2017). Hydrazine reduction, an essential aspect of methanotroph and anammox metabolism, requires considerable amounts of iron and may play a role in the movement of the metal through the EBPR environment (Maalcke et al., 2016; Fernández et al., 2020; Versantvoort et al., 2020). These biomechanical properties render EBPR systems potentially interesting for aquaponics given that alongside the augmentation of the macronutrient phosphorus, there is an unexplored potential for the remineralization of other plant-relevant nutrients.

The reutilization of one industry’s waste products (aquaculture) as a beneficial input to another industrial production process (hydroponics) has made aquaponics into a posterchild for circular economies. The size of an aquaculture system determines the potential scaling of fish to plant production volumes based on waste nutrient availability. It also sets internal limits, without supplementation, on the ability to satisfy plant nutritional needs based on the availability of specific nutrients poorly represented in soluble RAS effluent (e.g., Fe, Mn, Zn, B, Mo, and Cu). While fish nutritional requirements are controlled via the external addition of feed, gauging plant nutritional needs in a coupled aquaponics system is more challenging due to nutrient dynamism across the aquaculture production cycle and across the plant lifespan, not to mention the complex physiochemical influences on nutrient bioavailability (Yogev et al., 2016; Gichana et al., 2018; Goddek and Körner, 2019). Furthermore, the role of the rhizosphere—and its importance in nutrient bioavailability and assimilation—remains poorly understood (Badri and Vivanco, 2009; Richardson et al., 2009; Chaparro et al., 2014; Garcia and Kao-Kniffin, 2018; Guyonnet et al., 2018; Jacoby et al., 2020), especially in the hydroponic context (Lee and Lee, 2015). The multifactorial increase in both diversity and abundance of the microbial ecosystem in aquaponic systems, as compared to hydroponic counterparts, has previously been discussed as an explanative factor for the discrepancy in fertilizer requirements between the two cultivation systems (Wongkiew et al., 2017; Bartelme et al., 2018; Ayipio et al., 2019; Joyce et al., 2019; Melo and Corney, 2019; Yang and Kim, 2020). None of the nutrient streams (commercial solution, soluble effluent, remineralized effluent, and soluble + remineralized effluent) received additional supplementation over the duration of the study. While it was not expected that this would achieve the maximal yield for any of the aquaponics treatments, it does provide an important perspective into the capacity of the RAS waste streams to supply nutrients to the hydroponics component. If this results in comparable *in situ* nutrient concentrations as determined by plant sap analysis, this may suggest that elevated aqueous nutrient concentrations are alone insufficient at improving agricultural quality and yield.

In these experiments, a novel solids treatment system remineralizing nutrients from fish solids into liquid fertilizer was developed capable of reducing C and N while preserving the trace nutrient profile. Lettuce was grown in four parallel circuits, containing an inorganic hydroponic nutrient solution, a traditional coupled aquaponics loop, and two treatments investigating the remineralization capacity of an in-line solids treatment system as an auxiliary source of nutrient to complement standard aquaponics (with and without coupling to a coupled aquaponics loop).

By simultaneously exploring aquaponics as a fertilizer production system as well as a waste treatment system, we examine the applicability of aquaponics as a value addition to freshwater RAS. Besides contributing to more efficient, resource-conscious fish and plant production, this study explores the concept of crop quality with respect to mineral nutrients. We demonstrate that nutrient excess does not necessarily improve nutrient bioavailability and thus may not translate into improved product quality. Nutrient concentrations in the greenhouse water supply were compared to plant sap analysis data, allowing for a detailed characterization of the capacity for each of the four treatments to satisfy their nutritional demands. This study is the first to assess the capacity of an aquaponics system to target mineral nutrient bioavailability in downstream agriculture through an in-line solids treatment system.

## Materials and Methods

### Experimental Design

**FIGURE 1:**
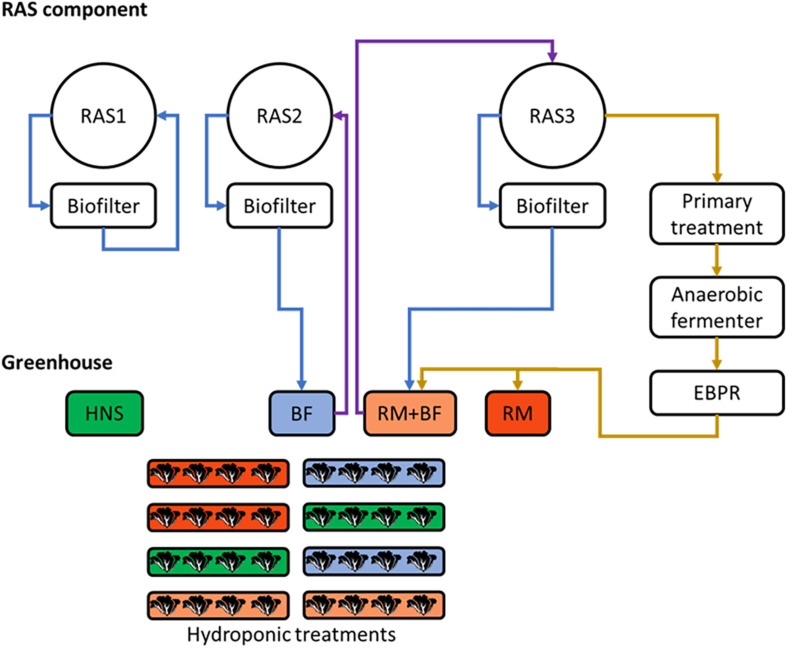
Schematic plan of the three parallel recirculating aquaculture system (RAS) units (left) and greenhouse (right). Blue arrows represent the transfer of wastewater toward the greenhouse, brown arrows represent the transfer of fish solids through the solids treatment pipeline, purple arrows represent the return flows from the greenhouse. Treatments were randomly assigned to their respective gutters.

An experimental aquaponics system was developed at INRAe-PEIMA (Sizun, France) to evaluate the performance of the solids treatment system within a fully functional aquaponics facility. The goal of this experiment was therefore to establish the boundary conditions for the commercial installation of this treatment system. The cultivation system consisted of three separate recirculating aquaculture system loops operating in parallel in three separate rooms (Figure 1). Nutrient solutions were diluted to their final concentration in the four wells within the greenhouse and automatically pumped through eight basins of five parallel nutrient film technique (NFT) gutters (Goponics, France) used to grow the lettuce.

Of the three RAS units, RAS1 ran independently, RAS2 was linked to the hydroponics treatment BF, and RAS3 was linked to hydroponic treatments RM and RM+BF. RAS2 was thus a traditional coupled aquaponics system, whereby oxidized water exiting the system’s biofilter was pumped through the corresponding hydroponics treatment before returning to the fish (BF). The biofilter effluent from RAS3 likewise circulated through the greenhouse, however, it was combined with the effluent from the solid waste treatment system (RM+BF). Effluent from the solids treatment system not mixed with biofilter effluent was stored in a separate well (RM). A commercially available hydroponics nutrient solution (Flora series; General Hydroponics, United States) was used as a control group (HNS), manually drained and replaced weekly. The Flora series consists of an N, P, and K heavy solution (FloraGro), a trace nutrient heavy solution (FloraMicro), and a third nutrient solution geared toward flowering, fruiting, and seed production (FloraBloom). Due to the variety of influences on the ultimate *in situ* nutrient concentrations in the plants, no additional nutrient supplementation was done apart from the treatment.

In this study, rainbow trout (*Oncorhynchus mykiss*) were raised from fry on site. Biofilters were set up for RAS 2 months prior to the addition of fish. An autochthonous lettuce cultivar well-adapted to the temperature and humidity profile of the region (Brittany, France) was chosen for this study and seedlings purchased from Tecnosem (France). Seedlings were transferred to the NFT gutters 3 months after fish cultivation began. The greenhouse unit, while not actively heated, was equipped with a thermometer and an automatic ventilation system that could keep the interior air temperature between 15 and 25°C throughout the cultivation period, with late-stage temperatures at the lower end of the range. Automatic pumping systems distributed nutrient solutions from the wells to the gutters.

### Design of the Solids Treatment System

**FIGURE 2:**
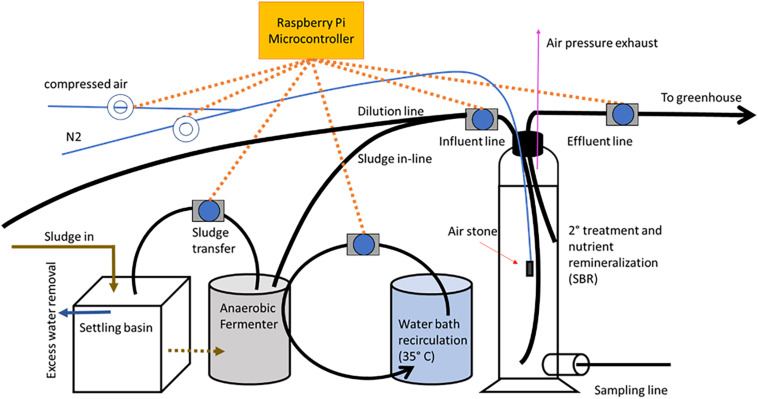
An overview of the solid waste treatment system.

The solid waste treatment system involved in the study consisted of a settling basin, an anaerobic fermenter, and a sequential batch reactor (Figure 2). Fish solids, passing into the settling basin directly from the RAS drum filter, were first concentrated in the settling basin with excess water evacuated via a lateral pipe that permitted water, but minimal solids, to pass through. A Raspberry Pi microcontroller was utilized to regulate the pumping of the fish solids from the settling basin into the anaerobic fermenter, then into the SBR, and finally from the SBR into the RM and RM+BF wells located in the greenhouse. The microcontroller additionally regulated the aeration through an air compressor and a nitrogen delivery system. The anaerobic digester was kept between 25 and 35°C by an adjacent water bath, with a pump recirculating water through tubing from the water bath, through the fermenter, and back into the bath in a closed loop. Prior to entering the SBR, the sludge was diluted 1:2 in water originating from the RAS sampling basin. This water, rich in ammonium, was chosen over sourcing from the aquifer to help balance the C:N ratio within the SBR.

The SBR itself consisted of a 3 L vessel with a main opening at the top, and a secondary lateral opening. That allowed a highly controllable environment where the dissolved oxygen (DO) could be maintained between 0 and 2 mg O_2_/L, and that regulated by the duration that either the compressed air or nitrogen lines were open.

To enrich the PAO proportion in the solid waste treatment pipeline, the SBR followed an alternating aerobic (DO = 2 mg/L) and anaerobic (DO = 0 mg/L) cycle. Due to the physical constraints of accessing the interior of the SBR, the DO, ORP, and pH over the course of the SBR cycle were calibrated externally and not monitored in real time. DO was thus set by measuring the shift during aeration with compressed air, or nitrogen gas, using a portable monitor. ORP proved to be a challenging parameter to measure, and thus was estimated through proxy based on the amount of bioavailable carbon entering the SBR. The SBR cycle was carried out as described in Table 1.

**TABLE 1:** Sequential batch reactor (SBR) cycling regime used in this study.

Phase	Action	Duration (s)	Description
1	Effluent	100	Evacuation of 1.5 L from the SBR
2	Influent	100	Import of anaerobic digester sludge diluted in RAS water totaling 1.5 L
**Anoxic phase**			
3	N2	60	Establishment of an anoxic environment
**Anaerobic phase**			
4	Still	1,240	Anaerobic fermentation
**Aerobic phase**			
5	Air	600	Aeration of the SBR
6	Still	300	Aeration turned off to keep DO from surpassing 2 mg/L
7	Air	900	Aeration of the SBR
8	Still	900	Shift toward starvation regime to promote P-release in PAOs

### Sampling

Sampling of aqueous nutrients was done biweekly for each RAS and the hydroponics nutrient solutions across the duration of the respective fish and plant cultivation periods. The pH in each RAS was regulated daily with NaHCO_3_ to maintain a pH of 7. Similarly, pH in the anaerobic digester was maintained at 7.5. Elsewhere, no modification was carried out as the pH remained stable and within acceptable boundaries. During sampling, ammonia, nitrite, nitrate, phosphorus (total phosphorus and phosphate), chemical oxygen demand (COD), and biological oxygen demand (BOD) were measured (Hach Lange, Germany).

In addition to *in situ* measurements, samples were assessed for plant relevant nutrients at harvest using commercial technology for greenhouse nutrient monitoring. This allowed a broad survey of nutrients in the RAS, solid waste treatment system, and hydroponics unit wells to be measured using inductively coupled plasma-mass spectroscopy (ICP-MS). Five hundred milliliter of each sample was sent for nutrient analysis with all handling was done in line with the NF EN ISO 5667-3 standard and specific analyses following other standard ISO protocols. Aqueous nutrient analysis was performed by Capinov SAS (France), while plant sap analysis was carried out by NovaCrop Control, Netherlands. Plant sap analysis was conducted on a pooled set of leaves from a specific treatment. Young leaves, old leaves, and roots were packaged separated after being collected 2 h after sunrise as per NovaCrop Control guidelines. All generated bioinformatics data were processed in Microsoft Excel and R.

Lettuce plants were harvested after 8 weeks of hydroponic growth. Fresh and dry shoot and root weights were measured, as well as root length, overall plant health, and total yield. ANOVA and Tukey multiples tests were used to determine whether treatments differed significantly in harvest parameters.

## Results

### Aqueous Nutrient Concentrations

**FIGURE 3:**
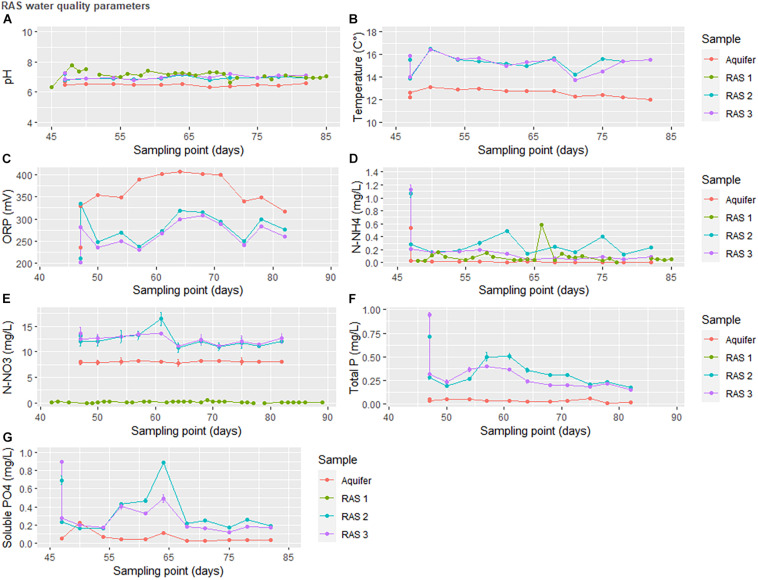
Water quality parameters in the recirculating aquaculture system at INRAe-PEIMA between the coupling of the recirculating aquaculture system (RAS) to the greenhouse (day 45) and the end of the experiment (day 81). RAS1 was operated as a traditional RAS, RAS2 ran as a traditional coupled aquaponics circulation system, and RAS3 contained both aqueous and solid waste treatment components.

*In situ* water quality measurements indicated that the stepwise oxidation of nitrogenous species in the RAS was relatively stable. Total phosphorus and phosphate did not exceed 1 mg/L but did increase following RAS coupling to the HP units, although by the end of the experiment concentrations returned to their original figures (Figure 3).

**FIGURE 4:**
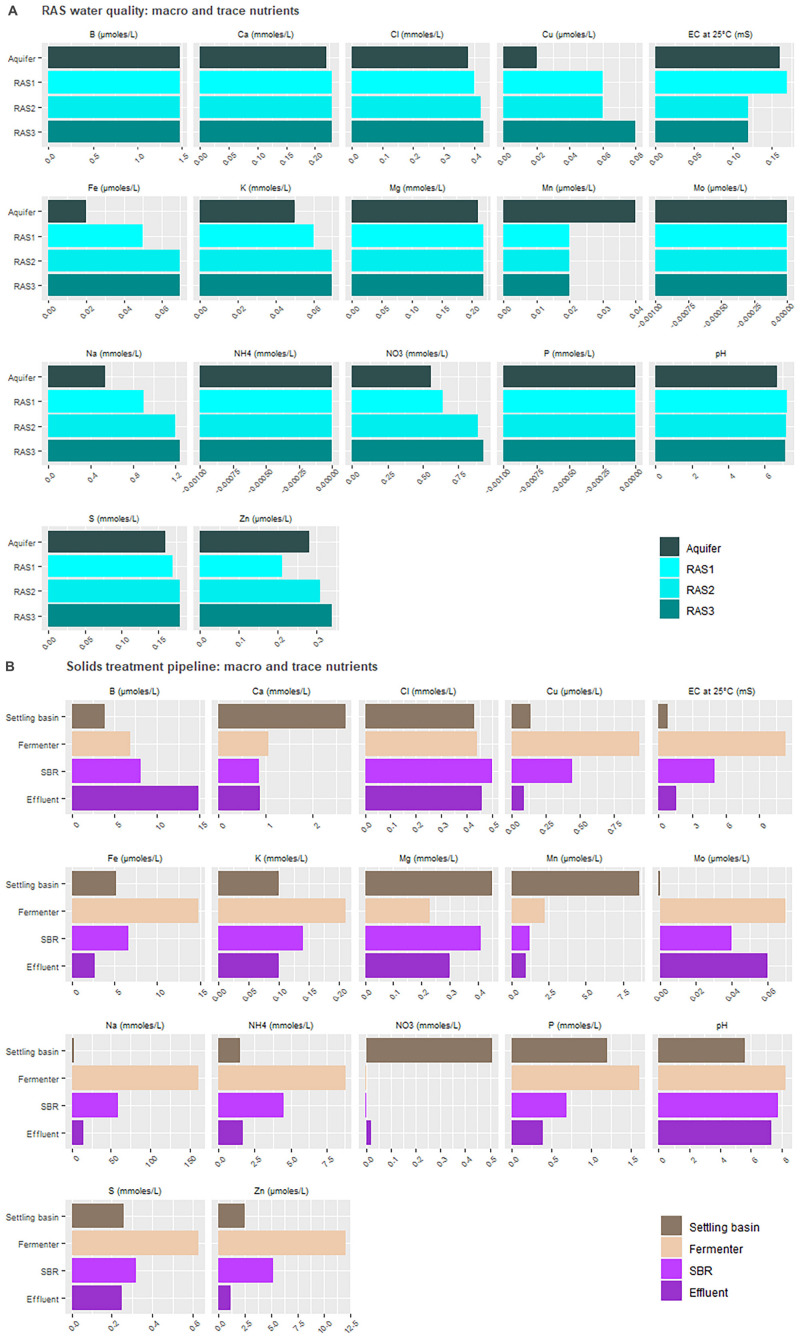
Nutrient load in the recirculating aquaculture system (RAS) **(A)** and solids treatment pipeline **(B)** at steady state conditions.

Aqueous nutrient concentrations indicated that many, but not all, essential plant nutrients were available in the water supply (Figure 4A). Unsurprisingly, virtually all nutrient concentrations in the output from the solid waste treatments (SBR) were elevated compared to the RAS water alone (Figures 4A,B). The notable exception to this rule was Mo. As uneaten feed was directed to the solids treatment system along with excreta, these data imply the nutrient is absent or minimally present in the feed. Charts for P, Fe, NH_4_^+^, and NO_3_^–^ mirror the shifts expected to occur in the reducing environment. Importantly, as the pH remained slightly alkaline, we can attribute the liberation of P and Fe to microbial activity and not acidic dissociation.

**FIGURE 5:**
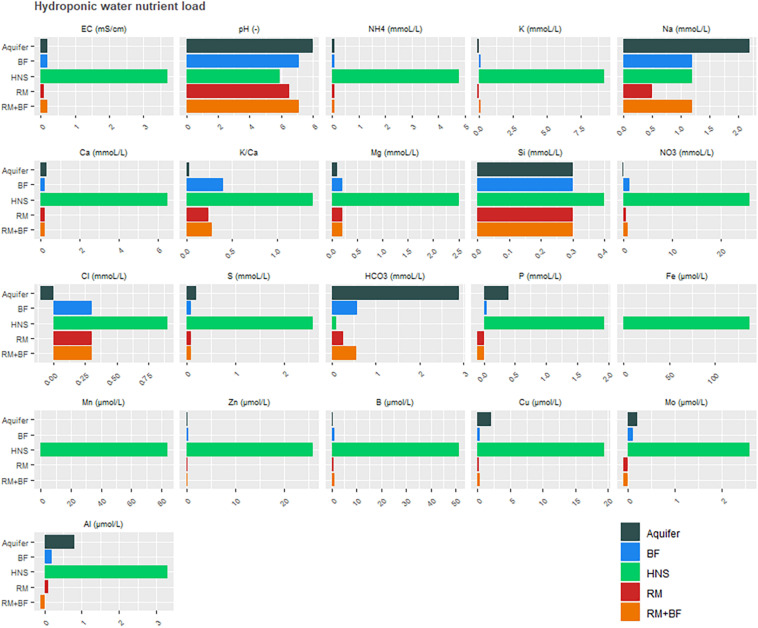
Nutrient loads across greenhouse nutrient solutions, all measurements were taken 1 week prior to harvest.

Nutrient composition in the greenhouse wells highlighted the elevated concentrations of virtually all plant-relevant nutrients in the commercial hydroponic nutrient solution (control), with the exceptions being Na, Al, and Si (Figure 5). Likewise, the pH across all nutrient solutions remained similar. Due to this high proportion of solutes, the EC of the HNS was proportionally higher. The N-NH_4_^+^ and N-NO_3_^–^ concentrations were much higher in the control solution than the coupled aquaponics solution.

### EBPR in the Aquaponics Context

**FIGURE 6:**
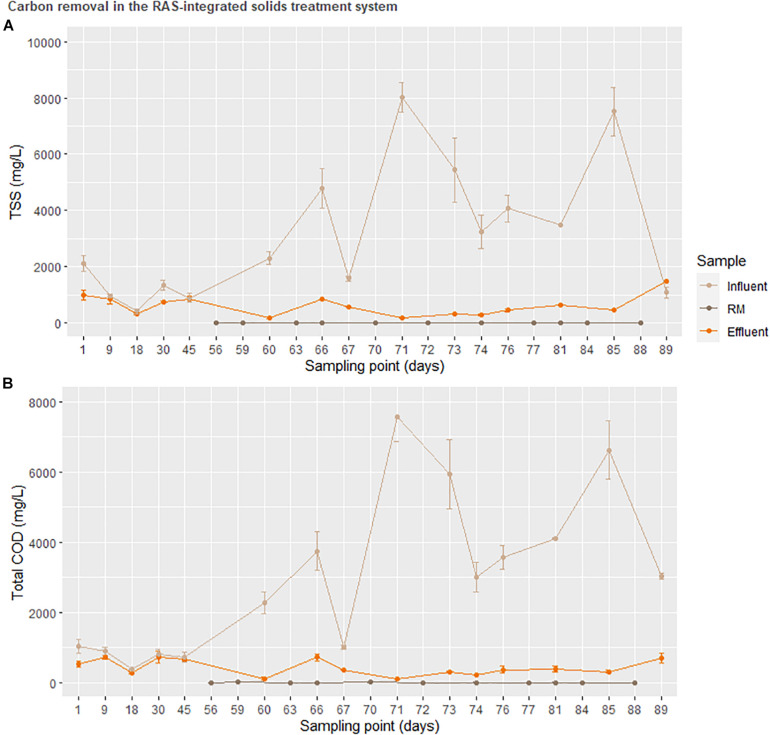
Total suspended solids and total carbon oxygen demand of sludge prior to entering the sequential batch reactor (SBR). The treatment RM was used to represent accumulation in a downstream hydroponic (HP) unit.

**FIGURE 7:**
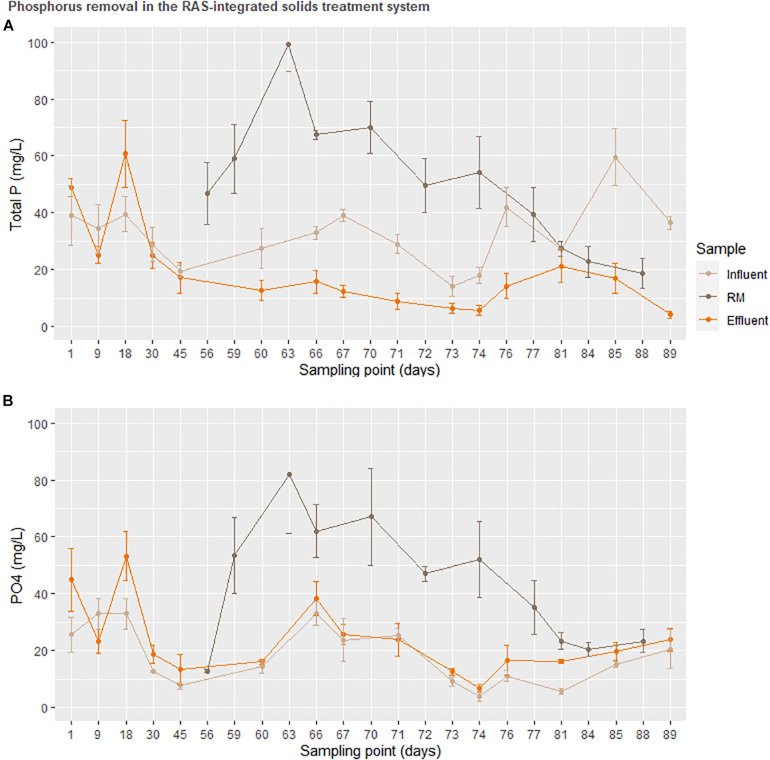
Total (top) and soluble (bottom) phosphorus remineralization in the solids treatment system normalized to total mass transferred. The treatment RM was used to represent accumulation in a downstream hydroponic (HP) unit.

Ultimately, the success of the solids treatment system in adjusting the concentration of several important plant nutrients (Figure 4B) alongside a drastic reduction in carbon (Figure 6) opens a unique niche for sustainable, cost-effective aquaponics cultivation. The adaptation of the enhanced biological phosphorus removal system into the aquaponics system necessitated two fundamental changes in the design. Firstly, the heterogeneity of the fish solids as a C-source did not permit an enrichment of the PAO strains as canonical to EBPR wastewater treatment systems. As the solids treatment system resulted in extensive reductions of the C and N load, this was considered an acceptable trade-off as a high C-load would be unsuitable for use as a liquid fertilizer. Secondly, rather than accumulating phosphorus in the granules as done in EBPR wastewater treatment systems, the operational procedure was modified to promote P-release in the granules immediately prior to effluent evacuation [starvation period following the aerobic phase (Table 1)]. Figure 7 suggests that most of the phosphorus leaving the system was soluble and accumulating in the downstream nutrient solution wells of the hydroponics unit. With respect to total phosphorus, a significant reduction over the duration of the solids treatment system indicated steady degree of extraction from the solids. Thus, while soluble P leaving the reactor was not significantly higher than the concentration entering the system (disproving the hypothesis), a net conversion of conjugated P to soluble P was evident. What is clear from Figure 5, however, is that the contribution of the solids treatment system to the phosphorus demand was low—indicating that this system would need to be scaled up before the nutrient demands from a greenhouse of this size will be met.

Nonetheless, the high degree of variation in influent COD was initially exacerbated by physical obstacles. These included clumping of the incoming fish solids, reduced flow in the tubing in part due to fish scale and mucous accumulation as well as biofilm growth, a problem that was later solved by diluting the influent sludge and prolonging anaerobic fermentation. Due to this practice, regular wasting of the SBR (removal of accumulated settled solids on the order of ca. 100 mg/week) is not represented in the graph. These problems are likely irrelevant at greater production capacities where fish solids are more bioavailable with a decreased proportion of scales and mucous, as well as larger piping diameters to handle greater flows (Lobanov et al., unpublished).

### Plant Nutrient Concentrations

The plant sap analysis was chosen as a tool to confirm the successful acquisition of nutrients by the plants from the surrounding aqueous milieu. Old and young leaves were sampled from the plants 2 weeks before harvest, as per standard NovaCrop Control protocols often used in the hydroponics industry to measure plant health. At harvest, old and young leaves were again sampled along with roots from the same plants to provide a comparative measure of nutrient distribution over time.

**FIGURE 8:**
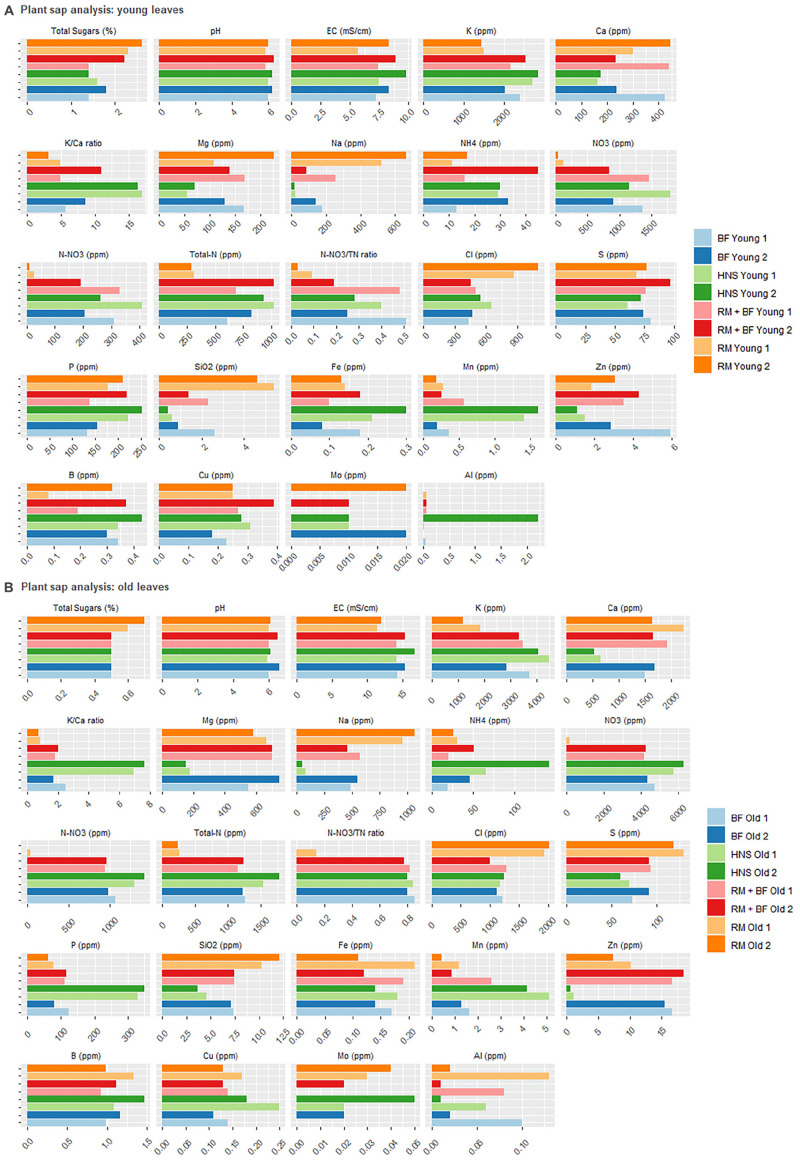
Plant sap analysis for young **(A)** and old **(B)** leaves collected 2 weeks prior to harvest and at the harvest.

**FIGURE 9:**
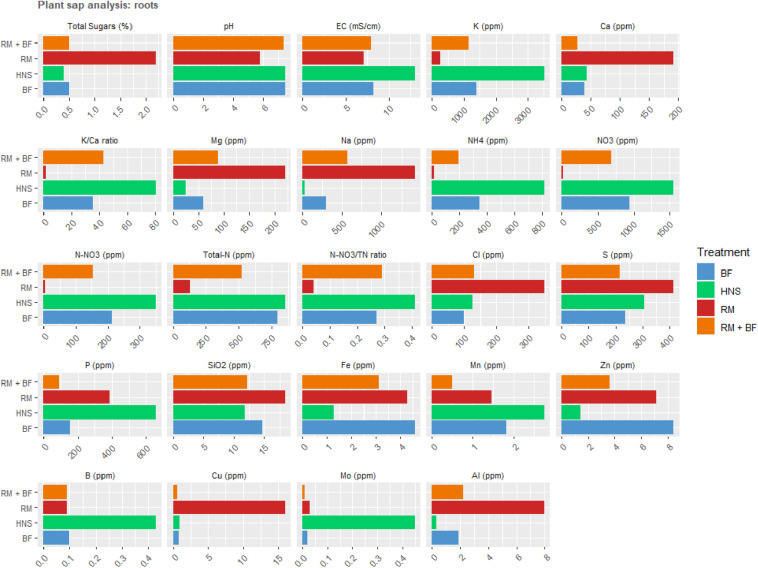
Plat sap analysis of the roots at harvest.

Of the physiochemical parameters, pH levels were constant for all three sample types: young leaves, old leaves, and root mass corresponding to Figures 8A,B, and 9, respectively. In terms of EC, all treatments were similar for young leaves, RM was slightly lower than the rest for old leaves, however, the HNS root EC was twice that of other treatments. In terms of sugar content, RM was an outlier with the highest percentage while other treatments averaged similarly together.

Plant nutrient concentrations varied drastically across treatments (Figures 8A,B, 9). Of the primary macronutrients, nitrogen (TN, NH_4_^+^, and NO_3_^–^) and phosphorus were more concentrated in the HNS control group than other treatments in old leaves and roots. N and P concentrations in young leaves were more balanced across all treatments, indicating that aquaponics-fertilized treatments could meet their nutritional needs but were not in excess of either nutrient. RM tended to be lowest in K, although HNS was significantly higher than other treatments only in the roots. Despite this, RM was the most balanced in terms of K:Ca, while HNS was heavily skewed toward K across all sample types.

For many nutrients, RM and HNS were opposite, with BF and RM+BF treatments falling in between. RM was generally higher in Ca, Mg, Na, Cl, S, SiO_2_, Cu, and Al although this was not universal for each nutrient at all sample types. The K/Ca ratio, often used as a general monovalent/divalent cation ratio, was most balanced in RM and most skewed toward K in HNS. Besides N and P, HNS had twice as much Fe in young leaves (0.255 ± 0.05 ppm vs. 0.158 ± 0.02 ppm for all other treatments). This was not the case for older leaves where all treatments were similar (averaging 0.158 ± 0.03 ppm) and was the opposite scenario in the roots (HNS = 1.29 ppm, RM = 4.27 ppm, BF = 4.59 ppm, and RM+BF = 3.13 ppm). It is thus difficult to correlate iron uptake efficiency to the treatment, however, it is clear that the nutrient rich solution did not result in consistently better uptake. In the water quality analysis (Figure 4A,B), Mo was not shown to be present in the RAS and solid waste treatment system but was present in the HNS control.

### Harvest

**FIGURE 10:**
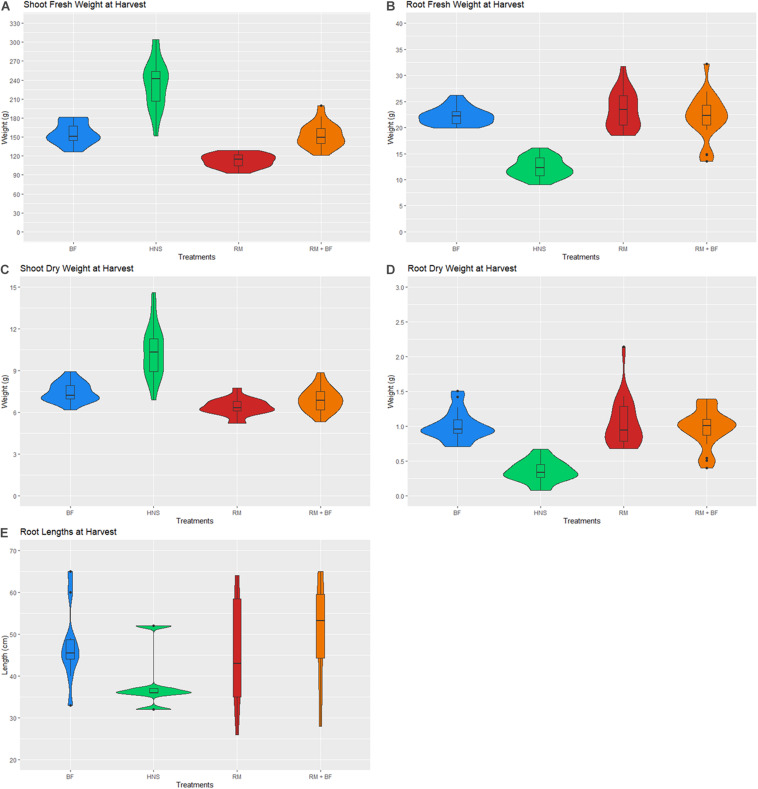
Lettuce head fresh **(A,B)** and dry **(C,D)** weights for shoots and roots at harvest. Root lengths are depicted in **(E)**.

The harvest was carried out after 8 weeks of cultivation as plants were beginning to crowd each other on the rafters. Lettuce heads and roots were weighed at harvest. Crop fresh and dry weight varied significantly across treatments, with the HNS control achieving the highest weight yield (Figure 10). Shoot yield varied most considerably across treatments, with the HNS treatment significantly larger than the others at *p* < 0.05 (Table 2). BF and RM+BF formed the next yield category, with the RM treatment trailing behind. While relatively abundant in micronutrients, the RM treatment was specifically deficient in N and K, likely responsible for the stunted growth.

**TABLE 2:** Tukey multiple pairwise-comparisons indicate that the hydroponic nutrient solution (HNS) crop was significantly different from other treatments in both shoot and root weights, although other treatments were more similar for certain metrics.

Multiples	Harvest parameter *p*-values adjusted to multiples				
	
	Shoot dry weight	Shoot fresh weight	Root dry weight	Root fresh weight	Root length
HNS-BF	0.00	0.00	0.00	0.00	0.49
RM-BF	0.02	0.00	0.95	0.68	0.98
RM+BF-BF	0.43	0.98	0.97	1.00	0.94
RM-HNS	0.00	0.00	0.00	0.00	0.62
RM+BF-HNS	0.00	0.00	0.00	0.00	0.24
RM+BF-RM	0.43	0.00	0.77	0.56	0.74

Root metrics reflected the nutrient saturation of the HNS treatment, with HNS root (fresh and dry) weighing about half of the other treatments. While root length was highly variable within each treatment, HNS similarly had the shortest.

A 1-way ANOVA suggests that all treatments were divergent in both shoot and root weights at *p* < 0.05. Removing the HNS treatment, BF, RM, and RM+BF diverged only in shoot dry and fresh weights but not in root mass. The Tukey multiple pairwise-comparisons test demonstrated that the HNS treatment was indeed the outlier, with the RM+BF and BF treatments being the most similar in harvest parameters (Table 2). The ratio between fresh and dry weights across all treatments and within each treatment is described in Table 3. Root lengths were not significantly divergent across any of the treatments as indicated by ANOVA and Tukey multiples tests.

**TABLE 3:** Ratio between fresh and dry weights for shoots and roots at harvest.

Treatment	Samples	Median fresh/dry	Mean fresh/dry
		ratio (%)	ratio (%)
All treatments	Root weight	4.31	4.22
BF		4.29	4.52
HNS		2.73	2.88
RM		4.04	4.49
RM+BF		4.54	4.4
All treatments	Shoot weight	4.71	4.76
BF		4.78	4.8
HNS		4.26	4.44
RM		5.52	5.65
RM+BF		4.61	4.53

### Disease Prevalence

With respect to disease, 16 of the 52 HNS lettuce heads had mold growth that developed shortly before harvest. No signs of disease were seen in other treatments. The treatment RM was severely deficient in nitrogen, likely explaining the yellowish coloration of the leaves commonly associated with a nitrogen-deficient state.

## Discussion

Although EBPR is a firmly established strategy for nutrient recuperation from municipal wastewater it has not yet been investigated in the context of solids treatment for freshwater aquaculture. This study is the first of its kind to assess the suitability of the technology as well as the impact of remineralization on the availability of trace nutrients in the downstream hydroponics unit.

### Balancing Macronutrient Excess With Micronutrient Deficiencies

Often, the concept of high-output yield (e.g., fast growth, with inherent economic implications) is prioritized over plant health. This view may need to be revised considering the relative abundance of trace nutrients in the lettuce across treatments. The chronic deficiencies present in the HNS control (Figures 8A,B, 9) suggest an internal triage response to manage the excesses of other nutrients, a phenomenon known as nutrient lockout (Du Laing et al., 2009; Enez et al., 2018; Pertiwi and Prastya, 2020). As reviewed by Marles (2017), several studies have indicated that the nutritional content of vegetables available to consumers has decreased in important mineral nutrients (e.g., Fe, Zn, Cu, and Ca) although the review stresses the lack of consensus surrounding potential causes (Marles, 2017). Nutrient-deficient vegetables is an issue of public health concern (Maggini et al., 2010; Keatinge et al., 2011). Understanding the differences between the hydroponic nutrient solution control and the aquaponics-derived nutrient streams can shed light on possible mechanisms underlying observed discrepancies in bioavailability.

### Comparing the Commercial Nutrient Solution to the Remineralization/Biofilter Effluent Solution

In comparing aqueous concentrations, it is evident that the control group receiving HNS had access to all relevant macro- and micronutrients at greater quantities in the water supply than was available for other treatments (Figure 5). Water originating from the RAS was deficient in Cu, Fe, Mn, and Mo, however, all of these elements were recovered from the solids treatment system (Figures 4, 5). One of the key findings of this work is the importance of incorporating a solid treatment system into aquaponics systems, that are traditionally reliant primarily on the dissolved nutrient fraction in the water that circulates between the fish and plants. The beneficial impact of integrating solids treatment into aquaponics cultivation were demonstrated by several researchers in the past (Delaide et al., 2018; Goddek et al., 2018; Khiari et al., 2019). Sharing fundamental objectives, the solids treatment system discussed in this study improves upon these systems by addressing the primary goal of nutrient remineralization alongside efficient waste treatment (C- and N-removal from bulk sludge to prevent eutrophication) alongside minimal endogenous biomass production. Previous studies, however, restricted their discussion of nutrient remineralization to macronutrients and a small number of micronutrients (Goddek et al., 2016b; Khiari et al., 2019).

With respect to the macronutrient phosphorus, it was assumed that approximately a third of the total system P would be carried downstream as soluble phosphate in the circulating water (Davidson et al., 2013; Cerozi and Fitzsimmons, 2017; Jaeger et al., 2019), and that the solids treatment system would further augment this quantity. However, no such trend could be observed in the nutrient solution wells (Figure 5). In terms of phosphorus, HNS, RM, and RM+BF had similar concentrations in young leaves. While soluble P was transferred to the greenhouse in the traditional aquaponics setup (BF), it appears that it was insufficient to meet plant needs. Surprisingly, water concentrations of P were nearly identical across RM, RM+BF, and BF treatments. It seems that the RM and RM+BF treatments were able to adapt to more efficiently acquire phosphorus, or perhaps that the phosphorus supplied by the solid waste treatment system was exceptionally bioavailable. The HNS treatment with 38.6 × more P than the other nutrient solutions had significantly higher P accumulation in roots and old leaves in addition to the slight gain in young leaves (Table 4). Ultimately, based on literature recommendations for lettuce sap P concentration (deficiency below 0.43% P/DW, sufficiency at 0.55–0.76% P/DW) we note that all treatments had their demand satiated (Barker and Pilbeam, 2015). Zn and Cu blocking, associated with an excess of P, seems to have affected the HNS treatment only in terms of Zn, where it was deficient (<1 ppm) in older leaves and borderline deficient in young leaves and roots (Forsee and Allison, 1944; Chapman, 1966; Cakmak and Marschner, 1987). No visible signs of Zn deficiency were observable, however (Barker and Pilbeam, 2015).

**TABLE 4:** Phosphorus as percentage weight over the average dry weight for each treatment.

	Old leaves	Young leaves
Treatment	% P	% P
BF	1.5	2.1
HNS	4.9	3.4
RM	1.0	2.8
RM+BF	1.7	2.6

The relationship between pH and nutrient bioavailability has long been a challenge in chemical fertilization as it quickly leads to unideal nutrient solubilities regardless the value. Below a pH of 6, Mn, Zn, and Fe become more soluble at the cost of Ca, Mg, and K. The pH of the solids treatment system did not strongly deviate from upstream or downstream components. While acidity in the RAS (pH 7.18 ± 0.04) dropped to 5.61 in the primary treatment, effluent leaving the pipeline had returned above 7, before stabilizing around 6.23 ± 0.5 across all hydroponic well measurements. From this we conclude that acidification was not responsible for the increased solubility of easily complexed nutrients such as P and especially Fe across the solid waste treatment system.

The overapplication of nitrogen (esp. nitrate) remains the most common detrimental impact of fertilizer misuse on crop health (Addiscott, 2005). Excess nitrate in plants leads to consistent disease symptoms such as excess intracellular moisture uptake, cell elongation, decreased total sugars content, and a weakening of the cell wall (Timmermans and Van De Ven, 2014). The commercial HNS was highly charged in both ammonium and nitrate (47 × and 30 × more concentrated than other treatments, respectively). While this disparity directly translated into higher ammonia and nitrate concentration in all plant sap samples, it led to only a slightly higher (1.7 ×) total nitrogen concentration, leaving N-NO_3_^–^/TN ratios to be similar to other treatments. Total nitrogen, calculated as the sum of inorganic and organic sources, is indicative of internal protein concentrations [about 85% of TN consists of protein (Barker and Pilbeam, 2015)]. Nitrogen needs were satisfied in all treatments except for RM, which displayed clear signs of N-deficiency both visually as described in the literature (Marschner, 1995; Barker and Pilbeam, 2015) and as well indicated plant sap analysis (Figures 8A,B).

Nitrate reductase requires Mo as a cofactor in the conversion of nitrates to amino acids, although it is difficult to assign a target threshold at which point this need is met. Barring potential blocking from other nutrients, this need appears to be satisfied when Mo nutrient solution concentrations exceed 0.06 mg/L as the case in across all treatments within this study. There was no significant difference in Mo concentrations in young leaves, however, HNS contained higher concentrations in old leaves and root samples (Rocha et al., 2020). None of the treatments appear to have suffered from Mo deficiency (<0.01 ppm).

Iron is also required along with Mo for healthy nitrogenase activity among other essential enzymatic functions (Marschner, 1995). While the hydroponic nutrient solution was 460 × higher in Fe than other nutrient solutions in our treatments, the extra supplementation resulted in only a 1.6 × increase (from 0.1575 ± 0.02 to 0.255 ± 0.05 ppm Fe) in young leaves, no significant difference in old leaves, and a decreased concentration in the roots compared to other treatments. It is important to note that we are unable to comment on the speciation (and thus the bioavailability) of iron. However, as the HNS control was prepared weekly and contained EDTA-chelated iron, we can at least maintain that iron was soluble and flowing through the roots. The reduction of iron from insoluble Fe^III^ to Fe^II^ occurs most commonly under anaerobic conditions. Recorded ORP values in the 200–400 mV range suggest that if this occurs, it is done in rhizospheric microenvironments or through the action of siderophore producing microorganisms (Barker and Pilbeam, 2015; Singh et al., 2019).

Magnesium is required for the production of chlorophyll at a Mg:N ratio of 1:4, and deficiencies can lead to nitrate hyperaccumulation (Schwartzkopf, 1972; Bousset et al., 2019). Despite the HNS having the highest concentration of Mg (14 × greater than found in other treatments), young HNS leaves were deficient for Mg (<100 ppm), although this was not the case for older leaves. K concentrations in the HNS treatment reflected the priority given to this nutrient by commercial fertilizers (92 × increase in the nutrient solution). RM plants, which had the lowest K concentration in young and old leaves, simultaneously had the highest total sugar percentage of all treatments, and thus were likely not symptomatic of K deficiency as described elsewhere (Haeder, 1974; Lawlor et al., 2004). The total sugars percentage is a widely used measure of plant health in terms of biotic and abiotic stress resistance. No treatments were considered deficient in total sugars (<0.5%), however, there was much variability across treatments and sample types.

The counterbalance of K against Mg, Ca, and Na is a well-established example of nutrient blocking (Schwartzkopf, 1972; Timmermans and Van De Ven, 2014; Barker and Pilbeam, 2015). The HNS treatment was richest in K across all sample types (young and old leaves, roots) while containing the least amount of Mg, Ca, and Na compared to all other treatments (Figures 8A,B). This contrasts heavily to the available concentrations of K (<0.1 mmol/L in other treatments, 9.2 mmol/L for HNS), Mg (14 × more concentrated in the HNS than other treatments), and Ca (29 × more concentrated in the HNS than other treatments), although Na was in a similar range (0.5–2.2 mmol) across all treatments. A relative low concentration of Ca in the HNS treatment was observed across all sample types, despite an abundance (29 × greater than other treatments) in the water supply, however, this was not below recommended values for lettuce (Barker and Pilbeam, 2015).

Chloride levels were similar across the HNS, BF, RM+BF treatments for sample type (although varied significantly across sample type). RM Cl concentrations (6.9 mg Cl/g DW), likely elevated as a reaction to nitrate deficiency, were well below toxicity thresholds (>23.0 mg Cl/g DW) (Johnson et al., 1957; Van der Boon et al., 1990). Sulfur was not deficient for any of the treatments, although the HNS treatment had the least across all sample types. While a known relationship between S and N concentrations has already been established, it is not well understood how N-excess impacts S metabolism; synergistic effects with P and K uptake have been suggested (Barker and Pilbeam, 2015). Boron aluminum, and copper were not deficient for any treatment. Silicon, widely associated with disease suppression (Rodrigues and Datnoff, 2015), was deficient in HNS young leaves, with low values reported for BF and RM+BF young leaves. No deficiency was seen in other sample types.

Of all the nutrients, Mn was definitively deficient (<1.2 ppm) in all treatments except for the HNS for young leaves, and at the limit of deficiency for RM lettuce in their old leaves and roots. As Mn deficiency is associated with retarded growth, this may have played a role in the yield discrepancy (Hannam and Ohki, 1988). However, none of the common indications of Mn deficiency were visible across any of the treatments (Barker and Pilbeam, 2015).

### Nutrient Concentration Comparison Between the Three Alternative Liquid Fertilizer Solutions

As the entire cultivation system (RAS-greenhouse coupling, with solids treatment) was in continuous operation, the hydroponic well nutrient concentrations (Figure 5) were considered representative of the concentrations that the plants were exposed to in the respective treatments. From this, we were able to determine the extent to which plants were able to satisfy their nutritional needs at these concentrations.

Condensing the above analysis across study results reveals a surprising set of trends. Firstly, all treatments were below the recommended threshold for iron, according to suggestions for instance by commercial companies routinely assessing hydroponic crop health (e.g., NovaCrop Control, Netherlands) who were used for the analysis and conduct testing for the well-established Dutch hydroponics industry. The topic of iron supplementation in aquaponics was reviewed recently by Kasozi et al. (2019), who highlighted the lack of consensus around optimal concentrations for vegetal and fruit-bearing plants (Kasozi et al., 2019). Nonetheless, it remains perplexing that the EDTA-chelated, highly concentrated, commercial iron solution was not capable of increasing vegetal iron concentrations. In the aquaponic systems, however, the story of iron is more complicated. On the one hand, iron was not detected in any of the three RAS, nor the aquifer environment (Figure 4). The anaerobic fermenter had elevated iron concentrations at levels that would have been sufficient for plant needs (0.83 mg/L), however, these concentrations were not maintained in the effluent nor nutrient solution wells. Ultimately, the similarity of all four treatments in the plant sap concentrations suggests that much work needs to be done in understand iron solubilization dynamics, whether the iron needs of plants are being satisfied, and how the rhizosphere can be better recruited to fulfill this demand.

RM was manly deficient in two macronutrients, K and N, as well as the micronutrient B. The BF lettuce alone were deficient notably in P, but as well B as per NovaCrop Control guidelines. All of these requirements were met in the RM+BF treatment, suggesting that the proposed solid waste treatment system has significant potential to address plant nutritional needs. The HNS control suffered from some unique deficiencies, namely of Mg and Ca in young leaves, as well as Na and Si in both young and old leaves. On the other side of the spectrum, all other treatments suffered from Mn deficiency. Thus, while iron supplementation remains an open question, Mn must definitely be supplied in aquaponics given the insufficient access to the micronutrient through the fish feed. Likely, there is an ideal nutrient supplementation level greater than the baseline concentrations established here. Whether this demand will be satiated by an expanded solids treatment system alone will need to be established in future studies.

### Sizing Up the Solids Treatment System to Match Aquaponic Needs

This study investigated whether an in-line, EBPR-inspired solids treatment system could improve nutrient remineralization while removing excess carbon and nitrogen from the system. These trends were demonstrable; however, it is likewise obvious that the efforts were insufficient to satiate all plant micro-nutrient needs.

On average, the solids treatment system resulted in a 12 × removal of total COD between the anaerobic fermenter (influent) and the hydroponics unit (effluent). Although this value does not account for solids removed from the system for SRT control, it is a considerable reduction. Considering the 450 kg tons of fish in the system producing ca. Forty-five kilogram dry weight solids with a theoretical average P of 23.9 mg/g dry weight (Schumann and Brinker, 2020), the solids treatment system encountered a theoretical P load of 1.08 kg. This resulted in ca. Forty-four milligram P was provided to the greenhouse daily from the ca. 1.85 g of sludge from the anaerobic digester passing through the SBR daily. While a P uptake requirement for the plants in not possible to define here, scaling up the SBR threefold would at least provide a daily discharge of around 150 ppm, a reasonable target concentration for plant P demand.

### Yields Comparison

Across all treatments, an average of ca. Ninety-five percent of the total weight (shoot and root) consisted of water. A notable exception to this rule were the HNS roots, which were ca. Ninety-seven percent water (Table 3). While from a mass yield perspective it is not desirable to increase the relative amount of root mass compared to marketable vegetal biomass, the essential role of the rhizosphere in plant nutrient acquisition and stress tolerance cannot be neglected. Rhizophagy has been identified as a principal mechanism for nutrient acquisition and microbial shepherding by plants, a topic that is well reviewed in current literature (Paungfoo-Lonhienne et al., 2013; White et al., 2018; Ameer and Hussein, 2020). In addition to nutrient uptake, endophytic microorganisms are now understood to be crucial to several fundamental plant functions (growth and development, oxidative stress reduction, disease and predation prevention) (Chaudhary et al., 2017; Patel et al., 2018; White et al., 2019). Plants that naturally grow in soil-less environments (e.g., bare rock) are particularly reliant on a diverse and well-developed endophytic community, which may suggest similar patterns in hydroponic cultivation systems (Burghelea et al., 2015; Remiszewski et al., 2016; Burghelea et al., 2018). Ignoring the role of the rhizosphere is ignoring a fundamental plant organ (Lynch and Whipps, 1990; Yang and Crowley, 2000; Lynch and de Leij, 2001; Richardson et al., 2009; Garcia and Kao-Kniffin, 2018; Guyonnet et al., 2018; Zhalnina et al., 2018). In this context, the differences in root length and mass between the control and other treatments suggest an underexplored contribution of the rhizosphere to nutrient uptake in hydroponic cultivation (Figure 10).

The impact of microbially-suppressing agrochemicals strongly diminishes and shifts the rhizosphere community, with effects on both the effective bioavailability of nutrients and the rhizospheric reserves available to plants (Rengel and Marschner, 2005; Chen et al., 2019; Singh et al., 2019; Huang et al., 2020). An inhibited exchange of organic acids and nutrients between plants and their rhizosphere has been shown to engender drastic effects on nutrient-recycling and secondary metabolites (impacting taste, antioxidant capacity, etc. of the crop). These changes have been described in soil systems although contextualization in the hydroponic context is lacking (Mozafar, 1993; Massalha et al., 2017; Garcia and Kao-Kniffin, 2018; Guyonnet et al., 2018; Singh et al., 2019). The onset of mold in nearly a third of the HNS treatment lettuce, but not in other treatments, suggests that even while the plants obtained a better mass yield, they were potentially compromised in other aspects. Whether this could be linked to nutrient deficiencies [e.g., Si deficiency in young leaves has been linked to increased disease susceptibility (Etesami and Jeong, 2018)] or whether it is the result of a diminished rhizosphere community, was not confirmed in this experiment, but is worthy of further investigation.

## Conclusion

Fundamentally, the challenge of closed environment agriculture is one of resource-use optimization. The exploitation of readily available, soluble aquaculture effluent expanded our conception of nutrient transfer in the hydroponic environment to include the role of microorganisms and the rhizosphere. Nutrient remineralization has not been adopted as unanimously mainly due to the challenges and carbon reduction and the additional costs associated with existing waste revalorization systems. This study contributes to the field by presenting a novel strategy for solids treatment to this base inspired from EBPR processes found in municipal wastewater treatment plants. This system permits simultaneous waste treatment (C- and N-reduction) with low residual biomass generation and a diverse trace nutrient spectrum for downstream hydroponics cultivation. To gauge the impact of the nutrient streams on agricultural yield and quality, we did not supplement for deficient nutrients. This strategy provided a unique perspective into the ability of the hydroponic crops to take up aqueous nutrients.

For this investigation, the micronutrient profiles of the remineralized effluent, traditional coupled aquaponics, and a commercial hydroponic nutrient solution were measured. Nutrient concentrations diverged significantly between the aquaculture-derived treatments and the commercial solution, which eclipsed other treatments for virtually every measured element in the water column. In contrast, plant sap analysis did not reflect a universally higher nutrient content in lettuce grown under excessive nutrient conditions.

Lettuce grown in the commercial HNS likewise experienced deficiencies of Mg and Ca (young leaves) as well as Na and Si (both young and old leaves). Uptake of certain elements (Cu, Fe, Mg, S, and Zn) was greater across aquaponic treatments than initially predicted, however, Mn was universally absent from aquaponic treatments. B and P were especially low in the standard aquaponics treatment (fertilization with soluble RAS nutrients only). Together this suggests that the solids treatment system in parallel to RAS soluble effluent may be advantageous for aquaponic facilities seeking to maximize the benefits of the fish solids for plant nutrition.

Nonetheless, iron remains the most capricious element to provide for plants. The evidence that neither the commercial solution, nor aquaponic treatments was wholly successful in increasingly iron uptake, suggest a need for future studies to determine minimal “optimal” concentrations for plants and as well the real repercussions of deficiencies on crop yield and nutritional quality.

## Data Availability Statement

The raw data supporting the conclusions of this article will be made available by the authors, without undue reservation.

## Author Contributions

AJ and VL developed the theoretical formalism of the study. VL carried out the experiment with support from LL, DC, and PP. VL performed the data analysis and wrote the manuscript with support from AJ. The final version of the manuscript received input from all authors. All authors contributed to the article and approved the submitted version.

## Conflict of Interest

The authors declare that the research was conducted in the absence of any commercial or financial relationships that could be construed as a potential conflict of interest.

## Abbreviations

BF: biofilter
CEA: controlled environment agriculture
DO: dissolved oxygen
EBPR: enhanced biological phosphorus removal
EC: electric conductivity
HNS: hydroponic nutrient solution
PAO: polyphosphate accumulating organisms
RAS: recirculating aquaculture system
RM: remineralization effluent
SBR: sequential batch reactor.
